# The Book Notices, Selections, Etc.

**Published:** 1859-09

**Authors:** 


☞ The book notices, selections, etc., of the present number of the Journal, have been prepared under the direction of W. Godfrey Dyas, M.D., Fellow of the Royal College of Surgeons, Ireland, late Demonstrator of Anatomy in the University of Dublin, etc.

Dr. Dyas is an accomplished scholar, an experienced editor and reviewer, and we deem ourselves fortunate in being able to announce that the same department will in future remain under his supervision.

